# The Extended-Sphenoid Ridge Approach: A New Technique for the Surgical Treatment of Skull Base Tumors in Pediatric Patients

**DOI:** 10.3390/brainsci13060888

**Published:** 2023-05-31

**Authors:** Roberto Garcia-Navarrete, Alfonso Marhx-Bracho, Javier Terrazo-Lluch, José Luis Pérez-Gómez

**Affiliations:** 1 Neurosurgery Department, National Institute of Pediatrics of Mexico, Ciudad de Mexico 04530, Mexico; marhxalfons@yahoo.com.mx (A.M.-B.); jaterrazo@gmail.com (J.T.-L.); jlperezgomez@gmail.com (J.L.P.-G.); 2 Neurosurgery Department, Naval Medical Center, SEMAR, Ciudad de Mexico 04470, Mexico

**Keywords:** skull base surgery, pediatric neurosurgery, craniopharyngioma, optic glioma, germinoma, Ewing’s sarcoma

## Abstract

The sphenoid ridge approach (SRA) was initially described as a surgical technique for treating vascular pathologies near the Sylvian fissure. However, limited studies have systematically explored the use of skull base techniques in pediatric patients. This study investigated an extended variation in the sphenoid ridge approach (E-SRA), which systematically removed the pterion, orbital walls (roof and lateral wall), greater sphenoid wing, and anterior clinoid process to access the base of the skull. Objective: This report aimed to evaluate the advantages of the extradural removal of the orbital roof, pterion, sphenoid wing, and anterior clinoid process as a complement to the sphenoid ridge approach in pediatric patients. Patients and Methods: We enrolled 36 patients with suspected neoplastic diseases in different regions. The E-SRA was performed to treat the patients. Patients were included based on the a priori objective of a biopsy or a total gross resection. The surgical time required to complete the approach, associated bleeding, and any complications were documented. Results: Our results demonstrated that the proposed a priori surgical goal, biopsy, or resection were successfully achieved in all cases. In addition, using the E-SRA technique was associated with a shorter operative time, minimal bleeding, and a lower incidence of complications. The most frequently encountered complications were related to dural closure. Conclusions: The extended sphenoid ridge approach represents a safe and effective option for managing intracranial tumors in pediatrics.

## 1. Introduction

In modern neurosurgery, minimally invasive skull base techniques are the cornerstone for treating lesions at the base of the skull [1].

The pterional approach is the gold standard technique for treating several pathologies of the skull base [2,3,4,5,6,7,8,9]. This approach offers the opportunity to treat lesions of the anterior and middle fossa and of the union of the medium and upper third of the clivus. A combination with other surgical techniques, such as the orbitozigomatic approach, allows for the treatment of neoplastic lesions that occupy large and complex skull base compartments [2,10,11,12,13].

Traditionally, minimally invasive techniques consider a careful dissection of the temporal fossa’s soft tissues to avoid the inherent risk of injury from handling brain tissue and cranial nerves. However, even in the most experienced hands, these valuable techniques require a relatively longer surgical time. In addition, they will be associated with non-negligible blood loss, pain, postoperative complications, and a longer recovery, in addition to the esthetic and functional sequelae that can occur—a loss of hair on the suture line on the skin, a higher frequency of defects in the skin surface of the temporal region secondary to the atrophy of the temporalis muscle, and temporomandibular joint dysfunction.

The sphenoid ridge approach (SRA) is a minimally invasive technique commonly used to treat pathologies near the Sylvian fissure [14]. It has been demonstrated to be safe and effective in treating aneurysms of the bifurcation of the internal carotid and middle cerebral arteries. Some isolated reports have suggested that an extended variation in this approach, involving the removal of the orbital roof, pterion, sphenoid wing, and anterior clinoid process, along with an extradural anterior clinoidectomy, can further expand the surgical field and provide access to the most profound areas of the skull base [15,16,17,18,19,20,21,22,23,24].

Pediatric patients lack formal descriptions of minimally invasive techniques for treating neoplastic lesions in their supraorbital, sellar, suprasellar, and parasellar regions. Hence, this report aims to describe the benefits of the E-SRA for managing skull base tumors in pediatric patients.

Therefore, in this study, we propose a comprehensive extended approach to the sphenoid ridge (E-SRA), which includes a centered craniotomy at the pterion, in addition to the removal of the orbital walls (roof and lateral wall), greater sphenoid wing, and anterior clinoid process. This extended technique aims to reach the most profound areas of the skull base.

## 2. Patients and Methods

From 1 March 2015 to 30 June 2020, a total of 115 patients with presumptive diagnoses of neoplastic lesions located in their supraorbital, sellar, suprasellar, and parasellar regions were identified from the Department of Neurosurgery at the National Institute of Pediatrics of Mexico. Among these patients, 36 were selected to undergo a biopsy or gross total resection by E-SRA. The biopsy cases were considered *a priori* when a suspicion of a visual pathway glioma and germ cell tumors did not show conclusive tumor markers in the preoperative evaluation. The E-SRA procedure was followed based on a collegiate decision made by the Neurosurgery Department. In addition, informed consent from the patients’ parents for the surgical procedure was obtained. The Ethics and Research Committee of the National Institute of Pediatrics evaluated and approved this study. 

The criteria for selecting patients for the E-SRA procedure were as follows: (1) no history of prior surgery involving the pterion region; (2) radiological evidence of neoplastic lesions with a solid component volume of less than 50 cm^3^; and (3) patients with neoplastic lesions suggestive of an optic pathway glioma. Patients who did not meet the criteria for the E-SRA were treated using conventional craniotomy and skull base surgery techniques.

**Figure 1:**
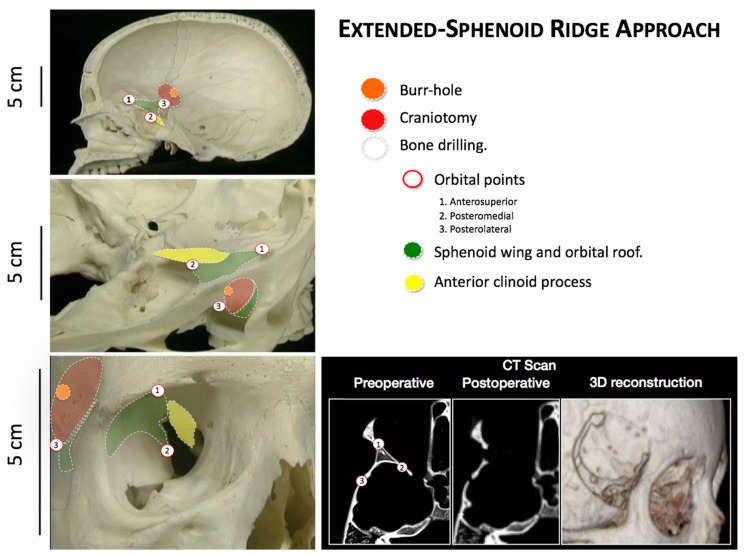
Surgical technique description of extended sphenoid ridge approach in pediatric patients.

E-SRA Technique: The SRA approach was performed as previously described [1]. A skin incision was made approximately 4 to 5 cm below the hairline and behind the external border of the eye, centered at the estimated location of the pterion. After the dissection of the skin and subcutaneous tissue, the superficial fascia and temporal muscle fibers were dissected along the skin incision. Next, a subperiosteal dissection was performed to expose the pterional region. A single burr hole was then made behind the pterion, followed by a small craniotomy (3 × 3 cm) around the visible landmarks of the sphenoid ridge. The hemostasis of the middle meningeal artery and dural vessels was achieved using a bipolar electrode after removing the bone flap. Once the pterion was removed, a triangular bone structure, defined by specific orbital points, became visible. The anterosuperior point, located at the upper and anterior angles, represented the union of the frontal bone and the internal table of the orbital roof. The posteromedial point, positioned at the medial and inferior angles, corresponded to the junction of the greater wing of the sphenoid bone and the base of the anterior clinoid process, projecting into the deep outer boundary of the superior orbital fissure. Finally, the posteroinferior point, found at the posterior and inferior angles, denoted the union of the internal table of the temporal bone and the wing of the sphenoid bone. The outer edge of the superior orbital fissure was delineated, and the meningo-orbital fold and meningo-orbital artery were dissected and coagulated to access the body and apex of the clinoid. The anterior clinoid process was drilled out and the optic strut was released at the lateral wall of the optic canal. At this stage, a dissection of the pretemporal dura allowed for an expanded surgical field of view, avoiding the use of brain retractors. Following the contour of the craniotomy edge, a dural opening was made, leaving a 5 mm free margin. After the tumor resection, the dura was sutured using a water seal technique with a 4–0 nylon suture. Sometimes, the use of a dural sealant reduces the risk of cerebrospinal fluid leakage. Next, the bone flap was repositioned and secured with non-absorbable sutures or mini plates. Finally, the temporal fascia and muscle were repaired using a 2–0 absorbable suture and the skin was closed with a 4–0 nylon suture. Subgaleal drainages were not utilized in our case (Figure 1 and Video S1). 

The surgical objective was determined a priori based on the surgical plan established during the Department of Neurosurgery’s session. The patients were categorized into two groups: those undergoing a biopsy and those undergoing an extensive resection surgery. Extensive resection surgery was considered when a neoplastic remanent accounted for less than 5% of the volume.

The evaluation of the surgical procedure included the length of the skin incision and the extension of the craniotomy, the time required to perform the E-SRA, and the associated bleeding. The complications were analyzed by reviewing all the clinical records until the patient was discharged from the institution and over a 24-month follow-up period. The statistical analysis presented the results as mean (±) standard deviation and ranges (lower and higher) for the quantitative variables and frequencies (%) for the qualitative variables.

## 3. Results

Patients and the Extended Sphenoid Ridge Approach Technique. We included 36 patients with a mean age of 7.8 ± 3 years; 17 male and 19 female patients were enrolled. The mean length of the skin incision was 4.2 ± 0.8 cm (3.4–5 cm) and that for the craniotomy area measured was 3.1 ± 0.5 cm^2^ (2.6–3.6 cm^2^). The average time taken to perform the E-SRA was 36 ± 6 min (30–42 min) and the quantified bleeding during the procedure was 20 ± 5 mL (15–25 mL). Brain retractors were not utilized in any of the cases and a dynamic retraction using cotton patties, bipolar forceps, and suction devices was preferred (Table 1).

Surgical objective. In all the patients, the objective of the surgery was accomplished: either a biopsy (22%) or a surgical excision (78%). A biopsy was performed in eight cases, when the intraoperative histopathological diagnoses were an optic pathway glioma (*n* = 4) or germinoma (*n* = 4). In 28 patients, an extensive surgical resection was performed (78%). It was considered total in 28 cases (86%) and subtotal (>90% volume) in 4 cases (14%) due to the invasion of diencephalic structures.

Histopathological diagnosis. The most common histopathological diagnosis was adamantinomatous craniopharyngioma (*n* = 22), followed, in order of frequency, by germinal tumors (*n* = 6), visual pathway gliomas (*n* = 4), pituitary adenoma (*n* = 3), and, in one case, orbital Ewing’s sarcoma (*n* = 1).

Complications. Vascular injury. No vascular injuries related to the approach were observed—specifically, injuries to the internal carotid artery during the anterior clinoidectomy. In one case, there was a vascular injury at the origin of the contralateral posterior communicating artery during the dissection of the tumor capsule. It was controlled by applying an aneurysm clip (Video S2). One patient with Ewing’s sarcoma presented a cerebrospinal fluid leak related to the infiltration of the dura mater above the orbital roof; two patients (5.5%) developed subgaleal hematomas in their temporal regions. No patient required further surgery to treat complications. Other than removing the orbital roof and lateral wall, we did not observe pulsatile exophthalmos. Despite the superior orbital fissure dissection, we did not observe clinical manifestations regarding cranial nerve paresis.

## 4. Discussion

The pterional approach, which was first described in the late 1970s, has become widely used for treating supratentorial neurosurgical diseases. It is considered to be the primary surgical corridor for accessing the skull base. In this study, we found that the extended sphenoid ridge approach (E-SRA) provided a safe and effective method for accessing neoplastic lesions in the skull bases of pediatric patients.

**Figure 2:**
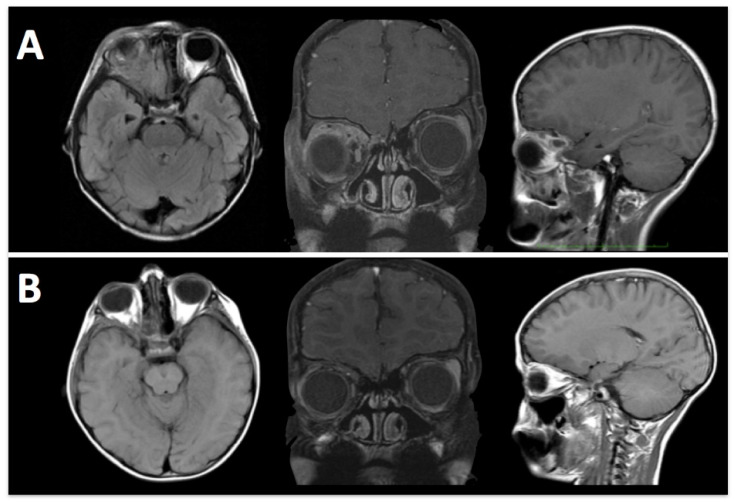
A 6-year-old boy was treated with orbital Ewing’s sarcoma suspicion. (**A**) Pre-operative MRI shows an extraconal lesion extended medially until ethmoidal cells. (**B**) Post-operative MRI confirms the removal of tumoral tissue.

By removing the orbital roof and lateral wall, the E-SRA technique allows for transcranial access to extraconal lesions, including those with an ethmoidal extension. This expanded access enables surgeons to reach tumors in challenging locations (Figure 2).

The E-SRA approach offers several advantages, including a shorter operative time, minimal bleeding, and a lower rate of complications. These benefits contribute to its improved surgical outcomes and patient recovery. However, it is essential to note that complications related to dural closure were the most frequently observed in our study.

**Figure 3:**
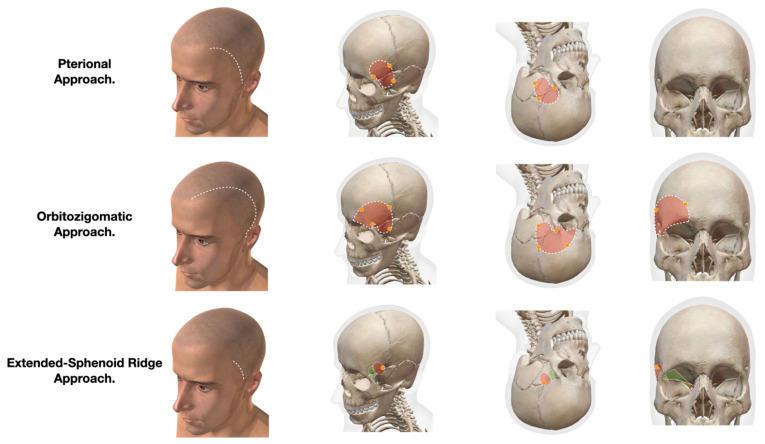
A graphic comparison of skin incision, craniotomy area, and orbital extension shows the benefits of the extended sphenoid ridge approach. (Modified models obtained from Atlas de Anatomía Humana Ver 2023.04.011. VISIBLE BODY^®^Argosy Publishing © 2007–2023).

Traditionally, various approaches, such as the supraorbital, subfrontal, pterional, and orbitozygomatic approaches, reach lesions in the sellar, suprasellar, and parasellar regions. However, these techniques pose specific risks to pediatric patients, including a longer surgical time, an increased bleeding volume, extensive soft tissue dissection, potential facial nerve injury, and a risk of temporomandibular dysfunction (Figure 3).

**Figure 4:**
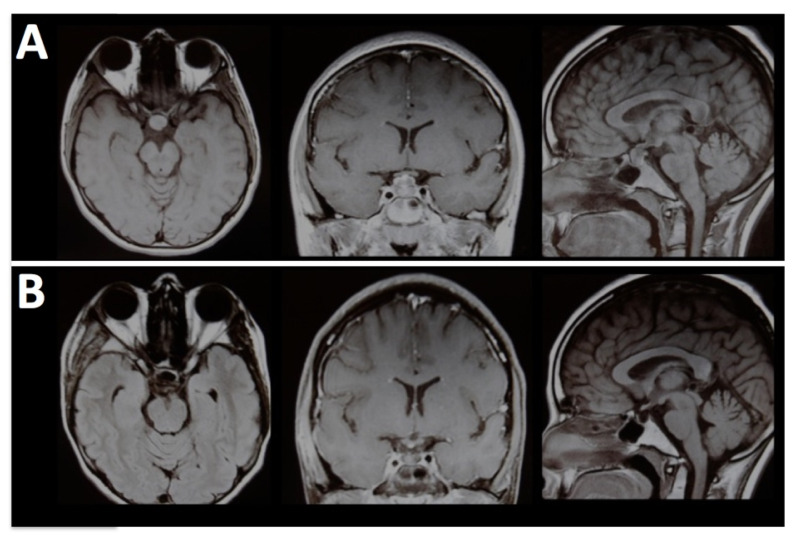
A 12-year-old girl admitted with diabetes insipidus and a growth delay. (**A**) Pre-operative MRI shows a sellar mass with extension to the infundibular stalk. (**B**) Postoperative MRI shows a gross-total resection.

In this study, the extended sphenoid ridge approach (E-SRA) provided comparable exposure with other skull base approaches, while minimizing the need for extensive soft tissue dissection (Figure 4).

**Figure 5:**
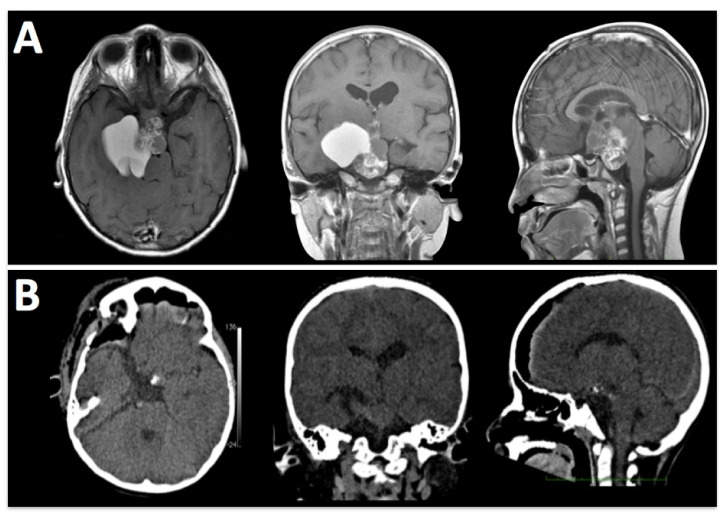
A 10-year-old girl with a history of headaches, visual disturbances, and panhypopituitarism. (**A**) Pre-operative MRI shows a mixed lesion with cystic and solid components. (**B**) A post-operative CT scan demonstrates the removal of tumoral tissue from sellar, parasellar, and suprasellar compartments. The histopathological analysis confirms the clinical suspicion of craniopharyngioma.

**Figure 6:**
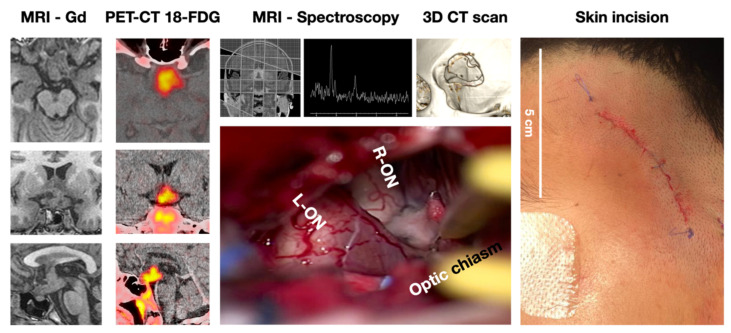
An 8-year-old girl with suspected optic pathway glioma was admitted. An E-SRA was performed and a sample biopsy was taken. The transoperative pathology study reported a germinal tumor. The image on the right shows the length of the skin incision behind the hair implantation.

The E-SRA technique allows for access to various structures in the skull base. The subfrontal corridor provides access to the olfactory nerves, optic nerves, inter-optic space, chiasm, lamina terminalis, A1 segment of the anterior cerebral artery, and anterior communicating artery. Dissecting Liliquist’s membrane allows for access to the interpeduncular cistern, posterior cerebral arteries, superior cerebellar arteries, basilar tip, and trunk. Figure 5 and Figure 6 and Video S2.

Understanding the complex dural arrangement in the pterional region is crucial. Our observations indicate that the meningo-orbital dural fold plays a critical role as a crossroad between the superior orbital fissure, pretemporal dura, and lateral wall of the cavernous sinus. A blunt dissection initiated at the meningo-orbital fold allows for a confident dissection of the dural sheath of the superior orbital fissure and the base and lateral aspects of the anterior clinoid process. In addition, removing the anterior clinoid process tip reveals the dural transition between the pretemporal dura and the lateral wall of the cavernous sinus in an anteromedial direction. These dural folds are relevant, as their early identification and dissection create a wide corridor to the cavernous sinus and its delicate contents. Thus, an adequate dissection of the meningo-orbital fold plays a crucial role in creating a wide corridor to access the orbital fissure, pretemporal dura, and lateral wall of the cavernous sinus (Video S1). 

Vascular damage, such as cavernous sinus or carotid artery injuries, was not observed during the E-SRA procedure. In one case, a vascular clip was applied at the origin of the contralateral posterior communicating artery during the craniopharyngioma’s capsule dissection.

When the approach to the sphenoid ridge was described, there was significant criticism regarding fixing complications related to vascular incidents. However, the video shows that it was possible to address this complication by placing an aneurysm clip at the origin of the posterior communicating artery. Clinically, the patient did not experience any functional decline, and subsequent imaging evaluations identified circulation in the affected vessel (Video S2). 

While transnasal endoscopic techniques help to treat tumors near the sphenoid sinus, their application in pediatric patients is limited and has specific challenges due to the poor pneumatization of the sphenoid sinus and an increased risk of vascular accidents. However, neuronavigation platforms can aid in directing a safe sphenoid bone removal to reach the sellar floor.

In conclusion, the extended sphenoid ridge approach is a safe and viable surgical option for treating intracranial tumors in pediatrics. This procedure should be considered among the available neurosurgical techniques, and our study provides a systematic description of skull base techniques for pediatric patients.

## Figures and Tables

**Table 1:** Demographic characteristics of patients treated with the extended-Sphenoid Ridge Approach.

Patient Characteristics.			
Age (Years)	7.8 ± 3		
Gender (Male/Female)	17/19		
Extended-Sphenoid ridge approach.			
Skin incision (cm)	4.2 ± 0.8		
Craniotomy area (cm^2^)	3.1 ± 0.5		
Bleeding (mL)	20 ± 5		
Complications			
	CSF Leakage		2.7%
	Subgaleal hematoma		5.5%
	Vascular injury related to the approach		0%
	Re-intervention		0%
Histopathological diagnosis and surgical outcome.			
Diagnosis	Volume (cm^3^)	Resection	
Craniopharyngioma (*n* = 22)	50 ± 12	Total Subtotal	81% (*n* = 18) 19% (*n* = 4)
Germinal tumor (*n* = 6)	25 ± 12	Total Biopsy	33% (*n* = 2) 66% (*n* = 4)
Visual pathway glioma (*n* = 4)	22 ± 12	Biopsy	100% (*n* = 4)
Pituitary adenoma (*n* = 3)	18 ± 12	Total	100% (*n* = 3)
Ewing´s Sarcoma (*n* = 1)	12	Total	100% (*n* = 1)

## Data Availability

No data is available due to privacy or ethical restrictions.

## Supplementary Materials

The following supporting information can be downloaded at: https://www.mdpi.com/2076-3425/13/6/888/s1, Video S1. E-SRA description. Video S2. Craniopharyngioma resection by E-SRA.

## Abbreviations

E-SRA	Extended Sphenoid Ridge Approach
SRA	Sphenoid Ridge Approach
